# The Relationship between Anxiety, Visual Function, and Symptomatology in University Students

**DOI:** 10.3390/jcm12206595

**Published:** 2023-10-18

**Authors:** Sonia Ortiz-Peregrina, Carolina Ortiz, Miriam Casares-López, Francesco Martino, Pilar Granados-Delgado, Rosario G. Anera

**Affiliations:** Laboratory of Vision Sciences and Applications, Department of Optics, University of Granada, 18071 Granada, Spain

**Keywords:** anxiety, mental well-being, university students, psychophysical visual test, vision, visual symptoms

## Abstract

Mental health concerns have emerged at the university level, with the psychological well-being of students being increasingly affected. This cross-sectional study investigated the proportion of university students having anxiety, and its effects on their visual function and symptomatology. We included 41 students (26.1 ± 4.8 years), and their visual function was assessed through several tests to produce a general visual performance index (VPI). The visual symptomatology was studied using the Conlon Visual Discomfort Survey and the Quality of Vision (QoV) questionnaire. The students were classified into two groups according to the Generalized Anxiety Disorder Screener (GAD-7) test (“no anxiety” and “anxiety” groups). The visual function evaluation indicated significantly worse VPI in the anxiety group (*p* = 0.047). These students also showed significantly higher scores in the Conlon survey (*p* = 0.004) and two subscales of the QoV questionnaire: symptom severity (*p* = 0.041) and symptom bothersomeness (*p* = 0.013). Moreover, the multiple linear regression model showed a significant association between visual discomfort according to the Conlon questionnaire and the level of anxiety (r = 0.405; R^2^ = 0.164; B = 0.405; *p* = 0.012). It is important to study the influence of psychological factors on vision, not only for refractive error, but also for binocular and accommodative disorders.

## 1. Introduction

In recent years, concerns have emerged about the mental health of doctoral students. High levels of academic demands and employment insecurity commonly lead to anxiety, depression, burnout, and emotional exhaustion among PhD candidates [1,2,3]. A Nature survey revealed that 36% of PhD candidates need help for anxiety or depression caused by their doctoral work [4]. On the other hand, mental disorders are also common among undergraduate students, but most of these seem to appear prior to their time at university. However, this condition is commonly untreated and causes them to drop out of their studies [5]. In the United States, one study reported that the incidence of diagnosed mental health conditions in college students increased from 22% to 36% between 2007 and 2017 [6].

The American Psychological Association defines anxiety as an emotional state characterized by feelings of tension and worry that lead to a series of physical changes (https://www.apa.org/topics/anxiety, accessed on 6 February 2023). The relationship between mental health and somatic disorders has been studied in health fields such as gastroenterology and neurology; however, there is a lack of knowledge in terms of ophthalmologic effects [7]. Sometimes, patients manifest visual or ocular symptoms not related to clinical findings. In this regard, Arntz et al., 2019, studied 39 patients with unexplained visual or ocular symptoms and found that all of them were suffering from psychological distress [7].

Continuous anxiety and stress elevate cortisol levels, causing an imbalance in the sympathetic branch of the autonomous nervous system (ANS), with negative consequences for the visual system [8]. The sympathetic branch of the ANS has been identified as playing a role in accommodation, having an inhibitory effect [9,10]. A recent study conducted in Spain investigated the incidence of anxiety and depression in 23,089 adults and the relationship of these conditions with vision and/or hearing impairments. The subjects were categorized as having vision loss if they mentioned distance- or near-vision difficulties. Thus, the authors found a relationship between anxiety or depression and visual loss [11]. Additionally, it has been reported that workers using video display terminals (VDTs) commonly report visual symptoms, and this condition seems to be related to anxiety, but not to visual acuity or refractive problems [12,13].

Despite a normal visual acuity and corrected refractive error, the consequences of sustained stress combined with prolonged periods of near work in students can lead to visual symptoms or visual discomfort, also known as asthenopia or visual fatigue [14,15,16]. This condition implies the appearance of unpleasant somatic symptoms and perceptual distortions, for instance, blurred vision, eye strain, or reading difficulties [14,15,17]. In this sense, it has been found that visual discomfort is a common problem among college students, and around 17% of this population may experience a moderate or high incidence of discomfort in their habitual tasks [14]. In many cases, these symptoms are associated with binocular or accommodation problems [18], so assessing visual function beyond refractive problems is of particular importance. Given the prevalence of mental health issues at university, the importance of near-vision tasks for students’ academic performance, and the possible consequences of this on visual function and visual complaints, the aims of the current study were threefold. Firstly, we aimed to investigate the proportion of students having anxiety in a group of undergraduates and PhD candidates. Secondly, we wanted to compare the visual function and symptoms of students with anxiety to those not suffering from this disorder. Thirdly, we aimed to investigate the relationship between the level of anxiety and some characteristics of the students, their visual function, and visual complaints, paying special attention to the factors that could be associated with the visual symptoms.

## 2. Materials and Methods

### 2.1. Participants

This cross-sectional study was conducted prior to the COVID-19 pandemic, and university students (PhD candidates and undergraduate students) were recruited. The undergraduate students were selected from those in their final year of studies to avoid any great age differences with the postgraduate students. Prior to the experimental session, the subjects’ refraction was checked. The exclusion criteria included having any ocular diseases, previous ocular surgeries, binocular or accommodation problems [18], as well as any medication being taken that could interfere with visual outcomes. The participants were also asked to abstain from alcohol consumption in the preceding 24 h and from caffeine-based drinks 12 h before the experimental session. Also, the sessions were undertaken on days when the participants had rested adequately the night before (at least 7 h of sleep). The students were grouped according to their anxiety level. For this classification, the Generalized Anxiety Disorder Screener (GAD-7) was employed, a validated test to identify possible disorders due to anxiety [19,20]. Scores between 5 and 9 indicate mild severity of symptoms and that the patient should be monitored. Scores between 10 and 14 are considered moderate, with a possible clinically significant condition. Finally, scores between 15 and 21 are considered severe and require active treatment. Spitzer et al. (2006) validated a cut-off of 10 points to identify clinically significant conditions, achieving good sensitivity, specificity and test–retest reliability [20]. Following this criterion, we established two groups, the no anxiety group (scores lower than 10) and the anxiety group (scores higher than 10).

This research was conducted in accordance with the Declaration of Helsinki and was prospectively approved by the University of Granada Human Research Ethics Committee (921/CCEIH/2019). A signed informed consent form was obtained from each participant prior to enrolling them in the study.

### 2.2. Visual Assessment

We conducted an extensive visual function evaluation using several tests and employing clinically standardized protocols [18]. All the visual tests were undertaken using the best optical correction. Vergence was evaluated by means of the near point of convergence, using reduced Snellen letters and measured with a rule. Accommodation was assessed using three tests: (1) binocular and monocular accommodation facility (AF) using flippers of ±2.0 D at 40 cm; (2) binocular and monocular accommodative amplitude (AA, push-up method); and (3) monocular accommodative response (AR). The accommodative amplitude was transformed into the difference of measured AA with regard to normal mean values for the subject’s age based on the Hofstetter formula [21]. In this, a positive value indicates a higher AA than expected for the participant’s age, and a negative value shows the contrary. The accommodative response was studied for three different demands (2.5D, 3D, and 5D) by means of an open-field autorefractor, the Grand Seiko WAM-5500 (Grand Seiko Co., Ltd., Hiroshima, Japan), a clinically validated refraction tool [22]. We employed the static mode, and the fixation target used was a 2 cm high-contrast black star (Michelson = 79%) printed on a white background. The subjects had to look at the target binocularly through a 12.5 × 22 cm open-field beam splitter, although the autorefractor is only able to record data from one eye at a time. We employed the same procedure as in previous studies [23], and nine measurements were taken for each accommodative demand. The accuracy of the AR was obtained as in Poltavski et al. [24], by subtracting the mean spherical equivalent of the nine measurements and the baseline refraction value (spherical equivalent) from the accommodative demand required by the target distance (2.5, 3, or 5D) [23,24,25].

Binocular and monocular high-contrast visual acuity (VA) was assessed at 5.5 m with the POLA Vista Vision Visual Chart System (DMD Med Tech srl. Torino, Italy), and the results were expressed in logMAR notation. Finally, ocular eye aberrations were measured using the WASCA Analyzer aberrometer (Carl Zeiss Meditec AG., Oberkochen, Germany), based on a Hartmann–Shack sensor. The aberration coefficients were computed for a pupil size of 5 mm. The total root mean square (RMS) of higher-order aberrations (HOAs) from the third to the fifth order were obtained. We included the following parameters: the spherical aberration Z(4, 0); the RMS for coma aberrations (computed as the square root of the sum of the squared coefficients Z(3, 1), Z(3, −1), Z(5, 1), and Z(5, −1)), and the trefoil (calculated as the square root of the sum of the squared coefficients Z(3, 3), Z(3, −3), Z (5, 3), and Z(5, −3)) [26].

As an overall measure of visual quality, the general visual performance index (VPI) was obtained and calculated from the individual visual parameters described in this section. The VQI was calculated by obtaining the z-scores for each visual parameter and averaging these. The Z-scores were converted when necessary, so that positive scores represented a better performance than the mean.

### 2.3. Questionnaires

The Conlon Visual Discomfort Survey was employed to assess subjective symptoms of visual discomfort [15]. This questionnaire comprises 23 items scored on a four-point scale as follows: 0 = event never occurs; 1 = occurs occasionally, a couple of times a year; 2 = occurs often, every few weeks; and 3 = almost always occurs. Thus, the total scores can range between 0 and 63 points. Furthermore, the items are divided into several symptom-related categories: soreness (3 items, total score 0–9 points); headache (2 items, total score 0–6 points); re-reading (3 items, total score 0–9 points); blur-diplopia (4 items, total score 0–12 points); movement-fading (9 items, total score 0–27 points); glare (1 item, total score 0–3 points); and slow reading (1 item, total score 0–3 points). Conlon et al. classified the participants who obtained scores from 0 to 24 as having low discomfort, from 25 to 48 as having moderate discomfort, and above 48 as having severe visual discomfort [15].

The Quality of Vision questionnaire (QoV) was also employed in this study [27]. This questionnaire is a validated instrument to subjectively assess visual quality in terms of visual symptoms such as glare, hazy vision, haloes, starbursts, blurred vision, double vision, distortion, and difficulties with focusing or depth perception. It consists of 10 items, each with three questions on the frequency, severity, and bothersomeness. The participants scored each item on a scale of 0–3. For the frequency subscale, each possible response was coded as: 0 = never, 1 = occasionally, 2 = quite often, and 3 = very often. For the severity subscale, each possible response was coded as: 0 = not at all, 1 = mild, 2 = moderate, and 3 = severe. Finally, for the bothersomeness subscale, each possible response was coded as: 0 = not at all, 1 = a little, 2 = quite, and 3 = very. The questionnaire was scored (Rasch-scaled) for each subscale on a 0–100 scale, with higher values indicating a worse quality of vision [27,28].

### 2.4. Statistical Analysis

All the statistical procedures were performed with the SPSS software v.26.0 (IBM Corp., Chicago, IL, USA), and statistical significance was set at *p* < 0.05. The normality of data distribution was checked using the Kolmogorov–Smirnov test.

Data obtained from visual tests for which it was possible to obtain monocular measurements were taken for a single randomly selected eye [29]. Visual parameter group comparisons (no anxiety vs. anxiety) were performed using the unpaired *t*-test or Mann–Whitney U test when normality could not be assumed. A Spearman correlation analysis was employed to study possible correlations between anxiety level and demographic data, visual performance (VPI), or visual symptoms (questionnaires).

Finally, a multiple linear regression model was employed to study which combination of independent variables (demographics, visual performance (VPI), or visual symptoms) could best explain the anxiety level according to the GAD-7 test. We used a forced entry method to select the independent variables, as we did not know the relationship between the independent and dependent variables from previous studies.

## 3. Results

**Figure 1:**
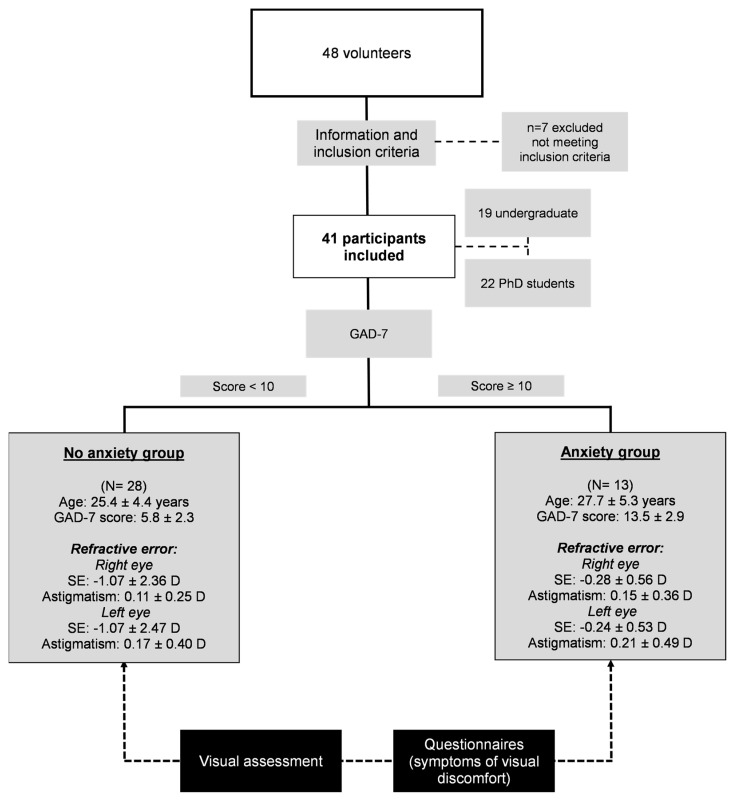
Flow chart of the study through the sessions (SE: spherical equivalent).

Of the 48 volunteers recruited, a total of 41 students were included in the study (7 males and 34 females), with a mean age of 26.1 ± 4.8 years. The group comprised 19 undergraduate students and 22 PhD students (Figure 1).

Using the GAD-7 score, 28 students were classified in the no anxiety group and 13 students in the anxiety group. The mean age ± SD was 25.39 ± 4.39 years and 27.69 ± 5.31 years for the no anxiety and anxiety groups, respectively, with the difference not being statistically significant (t = −1.460; *p* = 0.152). The anxiety group (N = 13) had a mean GAD-7 score of 13.46 ± 2.90, and the no anxiety group (N = 28) had a score of 5.82 ± 2.31 (Figure 1). This first group also indicated that they spent more hours doing near work tasks in a normal day (7.38 ± 2.41) than the no anxiety group (5.46 ± 2.76) (Z = −2.137; *p* = 0.033). Figure 1 also shows the refractive error data for both groups. The mean spherical equivalent was slightly more myopic in both eyes in the no anxiety group (−1.07 ± 2.36 D vs. −0.28 ± 0.56 D for the right eye and −1.07 ± 2.47 D vs. −0.24 ± 0.53 D for the left eye). However, statistical comparisons showed no significant differences (Z = −0.365; *p* = 0.772 and Z = −0.033; *p* = 0.989 for right and left eyes, respectively). Also, the astigmatism was very similar between the groups (0.11 ± 0.25 D vs. 0.15 ± 0.36 D for the right eye and 0.17 ± 0.40 D vs. −0.21 ± 0.49 D for the left eye) without significant statistical differences (Z = −0.019; *p* = 1.000 and Z = −0.570; *p* = 0.669 for the right and left eye, respectively).

### 3.1. Visual Performance Comparison between No Anxiety and Anxiety Groups

**Figure 2:**
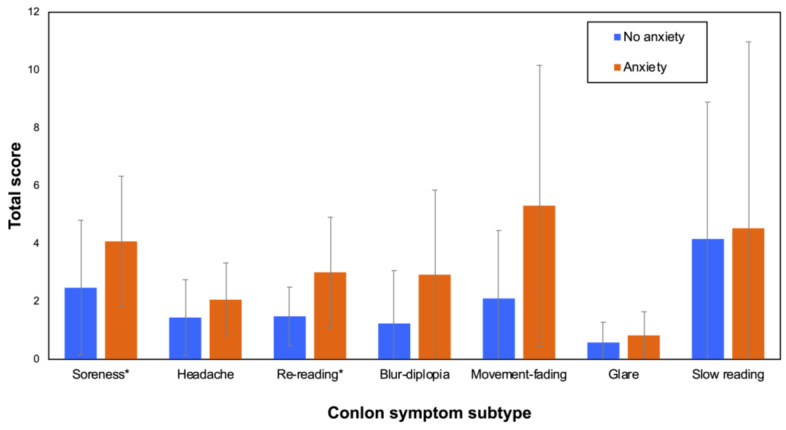
Comparisons of total scores (mean and SD) between the no anxiety and anxiety groups for the different symptom subtypes evaluated in the Conlon survey. The subtypes marked with an asterisk (*) indicate significant differences between the two groups.

Those students who demonstrated scores higher than 10 on the GAD-7 test (anxiety group) showed, in general, worse results in the visual parameters evaluated (Table 1). The visual parameters indicated statistically significant differences for monocular and binocular accommodative facility, monocular visual acuity, and the general visual performance index (VPI). Moreover, the anxiety group felt their quality of vision to be worse. For that reason, their scores for the QoV severity and bothersomeness subscales were significantly higher, as was the Conlon score, indicating more visual discomfort. Figure 2 represents the means and SDs of the total scores obtained for each symptom subscale in the Conlon survey. The group comparisons revealed significant differences in total scores between the no anxiety and anxiety groups for the soreness (Z = 2.129; *p* = 0.034) and re-reading (Z = 2.754; *p* = 0.007) subscales.

### 3.2. Associations between Anxiety Scores (GAD-7) and Demographics, Visual Performance Index (VPI), or Self-Perceived Visual Quality

To investigate the relationship between the level of anxiety (GAD-7 score) and the other aspects studied, a Spearman correlation analysis was used. The results showed that having a higher level of anxiety was not correlated to being an undergraduate or graduate student (rho = −0.100; *p* = 0.535), nor was it correlated to age (rho = 0.110; *p* = 0.493) or the number of daily hours of near-vision work (rho = 0.271; *p* = 0.086).

**Figure 3:**
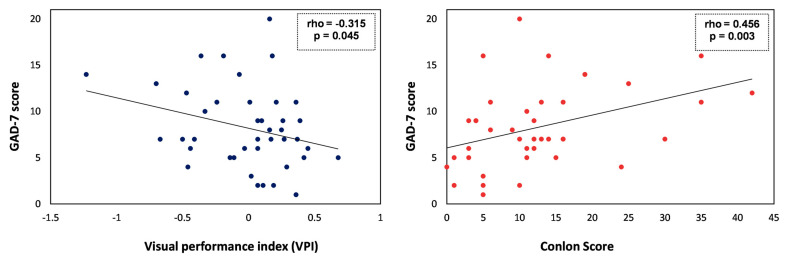
Scatterplots showing the relationship between GAD-7 score and visual performance index (VPI) (**left**) or and visual symptomatology (Conlon score) (**right**).

The VPI, a measure obtained from all the individual visual variables studied, did show a significant correlation with the level of anxiety. Thus, those students with higher scores on the GAD-7 (higher anxiety level) had a lower VPI, indicating a worse overall visual performance (rho = −0.315; *p* = 0.045) (Figure 3).

On the other hand, an association was found between higher levels of anxiety and more perceived visual symptomatology. Greater visual discomfort, as assessed by the Conlon questionnaire, was significantly correlated with a higher score on the GAD-7 (rho = 0.456; *p* = 0.003) (Figure 3). The same trend was found for the severity (rho = 0325; *p* = 0.038) and bothersomeness (rho = 0.414; *p* = 0.007) subscales of the QoV questionnaire, although no significant correlation was found with scores on the symptom frequency subscale (rho = 0.267; *p* = 0.091).

In order to analyze which combination of variables could show the strongest association with the level of anxiety (GAD-7), a multiple linear regression analysis was performed. As independent variables, we included demographic information (age, gender, type of student, and number of working hours a day), visual performance (VPI), and visual symptoms (Conlon score and QoV subscales score). From these, the model only selected the Conlon score as having a significant association with the level of anxiety according to the GAD-7 (r = 0.405; R^2^ = 0.164; B = 0.405; *p* = 0.012).

## 4. Discussion

Around 32% of the university students included in this investigation were classified as having anxiety according to the GAD-7. After comparing the no anxiety and anxiety groups, we found significant differences in certain visual parameters, such as the monocular visual acuity and monocular and binocular accommodative facility. However, the anxiety group indicated more visual complaints according to the Conlon survey and the Quality of Vision questionnaire. Significant correlations were found between higher levels of anxiety according to the GAD-7 test and a poorer visual performance (VPI), as well as with more visual symptomatology. Finally, the regression model showed that the anxiety level was significantly associated with the symptomatology score obtained by the Conlon test.

The alarming percentage of students classified as having anxiety according to the GAD-7 is similar to previous studies on PhD students [1,2,30], in which almost one third of the studied population had anxiety. This problem may be even greater currently, as the COVID-19 pandemic has increased the incidence of psychological problems in university students [31]. Over this period, a study in the Netherlands indicated that 47% of PhD students had an increased risk of developing mental disorders [32]. This represents an important public health concern, given that high levels of stress and anxiety over prolonged periods can cause psychosomatic disorders, which can manifest as physical alterations, including in the visual system [8]. In this regard, our visual function evaluation showed, in general, a worse performance for the anxiety group, as indicated by the significantly higher VPI. Specifically, the group with a higher level of anxiety demonstrated less binocular and monocular accommodative facility and worse monocular visual acuity. Continuous stress and anxiety can increase cortisol levels, affecting the sympathetic branch of the autonomous nervous system (ANS). It has been argued that the sympathetic ANS plays a complementary role in inhibiting the accommodative response [9,10,33]. Therefore, the mentioned effects of sustained anxiety in the sympathetic ANS could induce disaccommodation difficulties, decreasing the accommodative facility. We found no differences in accommodative amplitude (AA), nor in the accuracy of the accommodative response, possibly due to a primary control of the parasympathetic branch, which is not affected by increased levels of cortisol over time [8]. The decreased monocular visual acuity found in the anxiety group could also be due to difficulties with the relaxing accommodation. The role of sympathetic inhibition in accommodation seems to be augmented by concurrent near work or accommodative activity [34]. This would allow for adaptation to sustained near work tasks [35,36] by reducing the accommodative tone of the ciliary muscle, thus making it possible to dissipate the retention of the accommodative tone caused by long periods of intense near-vision work, which can lead to pseudo-myopia [9]. The anxiety group indicated prolonged periods of time doing near work (around 7 h/day), and this circumstance, together with the effects of stress and anxiety on the ANS, could explain the visual function results.

Although the objective data only showed accommodation effects in the anxiety group, these participants reported more visual symptoms. Nevertheless, accommodative facility has been demonstrated to be associated with visual discomfort [37]. The anxiety group had more visual discomfort according to the Conlon score, but also greater severity and bothersomeness according to the symptoms included in the QoV questionnaire. The correlation analysis showed that higher scores in the Conlon survey were associated with higher levels of anxiety. A previous study compared visual function in groups that demonstrated low and high levels of discomfort according to the Conlon survey. However, the authors found no differences in parameters such as the AA or AF [17], although they did find greater accommodation lags over time for those with high visual discomfort. Similarly, Chase et al. [38] reported a strong positive correlation in college students between discomfort symptoms (assessed through the Conlon survey) and accommodative lag for sustained fixation (30 min). Our results indicate that sustained anxiety or stress results in visual discomfort. Sustained stress can trigger psychosomatic symptoms, which could include visual symptoms [7,8]. In this context, a recent review discusses the relationship between stress and ocular disease. The authors conclude that although mental stress and anxiety are consequences of vision loss, they also cause this condition, proposing a new psychosomatic perspective with important implications for clinical practice [8]. Our findings agree with this assumption, given that students with higher anxiety levels notice visual symptoms to a greater extent (Conlon and QoV surveys), and this is associated with poorer overall visual performance (a worse VPI), as demonstrated by the correlations and regression analysis. In line with our results, other authors have also found a relationship between visual complaints and psychological factors. The study by Mocci et al. [13] found correlations between the presence of asthenopia (i.e., visual discomfort or symptoms) and different psychological factors associated with respondents’ work. The authors used different questionnaires to study a group of bank workers and found that aspects such as social support, group conflict, self-esteem, satisfaction, and the misuse of skills were associated with visual complaints [13].

While the Conlon survey assesses symptoms that could affect daily work (re-reading, headaches, soreness, etc.), the QoV questionnaire looks at other symptoms that could affect various tasks, including nighttime driving [14,15,27]. Both aspects are important and play an important role in our daily activities. For these reasons, apart from the psychological well-being of students, it is important to pay attention to their visual status and symptomatology, given that visual health and comfort could be compromised in relation to prolonged periods of stress and intense near work. In this sense, Filon et al. [12] studied 3054 public employees, finding a significant association between visual fatigue and self-perceived anxiety, psychological factors, and the use of electronic devices. However, the authors did not find a relationship between visual fatigue and visual acuity or refractive disorders [12]. Visual function encompasses aspects other than visual acuity and refractive status, such as accommodation, which plays a key role in near-vision work. Our results, together with those of other authors, indicate that the psychological state is important in terms of perceived visual quality; however, it is necessary to study vision in depth, not forgetting the accommodative or binocular system and its relationship with symptomatology. It is important to mention that within the anxiety group, the majority of subjects showed a moderate symptom score (up to 15 points). This, together with the small size of the group, requires further studies in subjects with severe anxiety symptoms. This could confirm our findings and show whether the association of anxiety with poorer visual performance is stronger. Finally, it is important to emphasize that when assessing people with anxiety disorders, in addition to considering the visual examination of the accommodative and binocular system, it is also important to evaluate the associated symptomatology. Questionnaires can be a good tool for this purpose, allowing us to obtain information on the precise situations and tasks in which these subjects experience visual discomfort. In this way, we will also be able to give recommendations to improve visual hygiene and ergonomics in such tasks or situations.

## 5. Conclusions

University students with GAD-7 test scores above the cut-off that could represent a possible anxiety disorder demonstrated a worse visual performance (VPI), with a significantly worse accommodative facility and monocular visual acuity. These results may be explained by longer periods of near work, as well as the consequences of stress and anxiety over time on the sympathetic branch of the ANS, which could lead to an inhibitory effect on accommodation. Similarly, the anxiety group reported more visual complaints in the Conlon survey and the QoV questionnaire, indicating a greater impact of the visual symptoms and complaints. The correlation analysis pointed to an association between the level of anxiety (GAD-7 score), visual performance, and visual complaints. In conclusion, an important proportion of the students in our sample may have anxiety, and this same group demonstrated more visual complaints and worse visual quality. To better understand the influence of psychological factors on the visual complaints of these students, it is important to assess not only the visual acuity or refractive error but also binocular and accommodative disorders, also taking into account the associated symptomatology.

## Figures and Tables

**Table 1:** Comparisons of visual parameters between no anxiety and anxiety groups.

	No Anxiety Group (N = 28)	Anxiety Group (N = 13)	Statistic t/Z	*p*-Value
Near point of convergence (cm)	6.06 ± 1.32	6.76 ± 2.22	−1.252	0.218
Binocular AF (cycles/min)	9.92 ± 3.46	7.14 ± 3.98	2.265	**0.029**
Monocular AF (cycles/min)	11.00 ± 3.97	7.56 ± 4.61	2.436	**0.020**
Binocular AA relative to age (D) (AA− AA (age)) *^,^ª	2.41 ± 2.05	0.95 ± 2.71	−1.240	0.224
Monocular AA relative to age (D) (AA− AA (age)) ª	1.29 ± 1.93	0.72 ± 2.92	0.748	0.459
Lag (2.5 D) *	−0.86 ± 0.50	−1.00 ± 0.63	−0.662	0.515
Lag (3 D)	−0.97 ± 0.63	−1.11 ± 0.68	0.628	0.534
Lag (5 D) *	−1.40 ± 1.02	−1.65 ± 1.25	−0.492	0.628
Binocular VA * (decimal scale)	1.26 ± 0.15	1.17 ± 0.18	−1.591	0.121
Monocular VA * (decimal scale)	1.14 ± 0.18	0.98 ± 0.17	−2.499	**0.012**
RMS total HOA *	0.20 ± 0.06	0.17 ± 0.07	−1.665	0.096
RMS Spherical	0.06 ± 0.07	0.02 ± 0.06	1.733	0.091
RMS Coma	0.12 ± 0.06	0.09 ± 0.06	1.845	0.073
RMS Trefoil	0.10 ± 0.05	0.09 ± 0.05	0.827	0.414
General Visual Performance Index (VPI) *	0.07 ± 0.32	−0.20 ± 0.43	−1.989	**0.047**
QoV Symptom Frequency Subscale	18.93 ± 12.44	27.18 ± 14.65	−1.758	0.094
QoV Symptom Severity Subscale *	19.76 ± 13.24	29.23 ± 15.22	−2.056	**0.041**
QoV Symptom Bothersomeness Subscale	14.88 ± 11.95	29.49 ± 17.15	−2.774	**0.013**
Conlon Total Score *	9.44 ± 7.06	19.23 ± 11.75	−2.849	**0.004**

* Mann–Whitney U test. Significant *p*-values are highlighted in bold. ª positive value indicates a higher AA than expected for the participant’s age. Abbreviations: AF, accommodative facility; AA, accommodative amplitude; VA, visual acuity; RMS, root mean square; HOA, high-order aberrations; QoV, quality of vision.

## Data Availability

Available from the corresponding author on reasonable request.
